# The Repercussions of Amyotrophic Lateral Sclerosis on the Orofacial Sphere: A One‐Year Prospective Longitudinal Study

**DOI:** 10.1111/scd.70170

**Published:** 2026-04-08

**Authors:** Vincent Vaudroz, Annemarie Hübers, Stavros Kiliaridis, Gregory S. Antonarakis

**Affiliations:** ^1^ University clinics of dental medicine, University of Geneva Geneva Switzerland; ^2^ Department of Clinical Neurosciences Clinic of Neurology, Geneva University Hospitals Geneva Switzerland; ^3^ Clinic of Neurology, University Hospital, Heinrich Heine‐University Düsseldorf Germany; ^4^ Department of Orthodontics and Dentofacial Orthopedics Dental School/Medical Faculty, University of Bern Bern Switzerland

## Abstract

**Aim:**

*The aim of this longitudinal study was to evaluate the repercussions of amyotrophic lateral sclerosis (ALS) on orofacial function, dental health, and the development of malocclusions, in order to assess whether disease progression influences oral and craniofacial outcomes*.

**Subjects and methods:**

Thirteen patients diagnosed with ALS according to the Gold Coast criteria were enrolled to be examined at two time points (T1 and T2), with a one‐year interval. The ALS Functional Rating Scale‐Revised (ALS‐FRS‐R), the Nordic Orofacial Test Screening (NOT‐S), the Decayed Missing and Filled Teeth (DMFT) index, Plaque Index, and standard orthodontic assessments were used to quantify changes in disease progression, orofacial function, dental health, and occlusal parameters, respectively. Statistical evaluation: Paired sample *t*‐tests were performed to evaluate differences between T1 and T2 for continuous variables. Chi‐square and Fisher's exact tests were used for categorical data. Multiple linear regression analyses were carried out to assess potential associations between general disease progression, ALS type, and orofacial functional or dental health decline. A significance level of *p* < 0.05 was adopted for all analyses.

**Results:**

Thirteen patients were examined at T1, 10 of whom completed both evaluations. A significant deterioration in the general disease condition was observed (ALS‐FRS‐R: mean difference −6.0 ± 6.98; *p* = 0.024). Orofacial function worsened significantly as reflected by an increase in NOT‐S total score (+2.3; *p* = 0.001). Dental health also declined, with a significant increase in DMFT (+1.8; *p* = 0.014) and Plaque Index (+0.4; *p* = 0.004). However, occlusal parameters remained stable over the 12‐month period, with no significant changes in overjet (*p* = 0.860) or overbite (*p* = 0.347). The bulbar type of ALS seems to show worse deterioration of orofacial function over time, and individuals with more significant general disease progression also showed worse orofacial functional decline.

**Conclusions:**

ALS has a significant impact on orofacial function and dental health, characterized by neuromuscular deterioration, increased plaque accumulation, and a higher number of affected teeth. Despite this decline, dental occlusion appears to remain stable in the short term. These findings highlight the need for interdisciplinary and preventive oral care strategies in the management of patients with ALS, aiming to preserve oral function and quality of life in a progressively disabling disease.

## 1 INTRODUCTION

Amyotrophic lateral sclerosis (ALS) is a progressive neurodegenerative disorder that affects both upper and lower motor neurons, making it one of the most debilitating diseases of this type. It involves the degeneration of motor neurons in the primary motor cortex, corticospinal tract, brainstem, and spinal cord, leading to a loss of mobility followed by muscle atrophy. The disease is characterized by a selective loss of motor neuron function and is divided into two forms: the spinal‐onset form (60%–70% of cases), typically corresponding to sporadic ALS (90%–95% of all ALS cases), and the bulbar‐onset form (20%–30%) [1, 2].

The spinal‐onset form typically begins with muscle weakness in the limbs, often starting in one limb and gradually spreading to others. This form is associated with a later onset of respiratory complications and may have a somewhat more prolonged progression. In contrast, the bulbar‐onset form, which begins in the muscles involved in speech, swallowing, and chewing, generally leads to earlier onset of difficulties such as dysphagia and dysarthria, progressing more rapidly [3, 4]. The predominance of the spinal form underscores the importance of evaluating oral health across the broader ALS population.

The incidence of ALS ranges from 1 to 2.6 cases per 100,000 people annually, with a prevalence of about 6 per 100,000 [3, 4]. This rare disease typically manifests around the ages of 58 to 60. Most individuals with ALS die from respiratory failure, which occurs within 30 to 36 months of diagnosis in 50% of patients. For those who surpass this threshold, the majority tend to pass away within 5 to 10 years post‐diagnosis [5].

From a dental and orofacial perspective, the bulbar form particularly impacts chewing, swallowing, salivary control, tongue mobility, and oral hygiene, which collectively increase oral‐health risks. Dysphagia, highly prevalent in ALS, leads to weight loss, malnutrition, dehydration, and increased retention of food particles, thereby promoting dental caries and periodontal disease [6, 7].

Dysarthria frequently precedes dysphagia and reflects motor impairment of the tongue. Approximately 60% of patients exhibit tongue abnormalities, including weakness, atrophy, fasciculations, or coating, all of which may impair oral clearance, contribute to halitosis, and increase the risk of aspiration pneumonia [6, 8].

Reduced maximal mouth opening, decreased bite force, and difficulties with lateral or protrusive mandibular movements are commonly reported. Patients with ALS are generally not prone to bruxism, although mucosal injuries may occur [8].

Sialorrhea, mainly in bulbar ALS, results from impaired perioral and lingual control combined with reduced swallowing frequency, and is associated with risks such as aspiration, cheilitis, and sleep disturbances [9, 10, 11]. Conversely, xerostomia linked to polypharmacy or mouth breathing can worsen caries risk and oral discomfort [12].

Although no specific studies have evaluated malocclusion development in ALS, existing knowledge suggests that tongue posture alterations, facial muscle atrophy, macroglossia‐like presentations, and chronic mouth breathing may disturb orofacial equilibrium. Dental and orthodontic management of patients with ALS remains challenging, and tooth extraction is often the treatment of choice once dental problems arise, especially given the limited life expectancy [13]. ALS may potentiate periodontal pathologies, and active periodontitis can induce dental displacement and flaring and accelerate tooth loss [14]. With tooth loss, malocclusions may develop via tipping, rotations, and extrusions, but major changes may remain limited due to disease progression [14].

Similar mechanisms are observed in Duchenne muscular dystrophy and cerebral palsy, where altered muscle function and head posture are associated with posterior crossbites, anterior or lateral open bites, and Class II malocclusions [15, 16, 17]. Macroglossia‐like tongue posture may accentuate transverse discrepancies, spacing, or proclination of incisors [18]. Chronic mouth breathing may increase facial height, induce posterior mandibular rotation, and contribute to open bite and increased overjet [19].

To date, data are lacking regarding the influence of ALS progression on orofacial function, dental health, or the development or worsening of malocclusions. Given that patients with ALS represent a medically vulnerable population with complex oral‐care needs, this evidence gap is particularly relevant for special care dentistry. The present study aims to explore whether ALS is associated with disturbed orofacial functions, dental malocclusions, and the presence of dental pathologies over a 12‐month period, using a prospective longitudinal study design.

### 1.1 Materials & Methods

The present prospective longitudinal study was approved by the Cantonal committee for research ethics involving human beings (BASEC‐ID: 2020‐00509)

### 1.2 Sample

Thirteen patients with ALS, recruited from a single center, took part in this study, out of approximately 30 patients with ALS followed up at the University Hospitals of Geneva, Switzerland. All patients had been diagnosed with ALS according to the Gold Coast criteria [20] by an experienced neurologist. These criteria diagnose ALS by identifying progressive motor impairment associated with signs of upper motor neuron dysfunction (spasticity, hyperreflexia) and lower motor neuron dysfunction (fasciculations, atrophy) in at least one body region. Patients were classified as being affected primarily with the bulbar or spinal type of ALS. Recruited patients were planned to be examined at two time points, once at baseline (T1) and once 12 months after the baseline visit (T2). During these two visits, the general disease course, orofacial function, dental health, and malocclusions were evaluated. These examinations were carried out at the hospital, the University clinics of dental medicine, or at the patients’ homes, as per the preference of the patient and/or their family.

No a priori statistical power calculation was performed, as the primary objective was exploratory and constrained by the limited pool of eligible patients. The sample size, therefore, reflects the real‐world availability of patients with ALS able to undergo dental and orofacial assessments, which is a common limitation in rare and severe neurodegenerative diseases.

## 2 Methods

### 2.1 General Disease Course

The general course of the disease was assessed using the ALS Functional Rating Scale Revised (ALS‐FRS‐R). This clinical tool is used to assess ALS progression by measuring the patient's functional abilities. The questionnaire consists of 12 items, each scored from 0 (total loss of function) to 4 (normal function), covering various domains such as speech, salivation, swallowing, writing, eating, dressing, hygiene, mobility, and respiratory functions. Individual scores are summed to obtain a total score ranging from 0 to 48, with higher scores indicating better physical function [21]. This evaluation was conducted by an experienced clinical neurologist.

### 2.2 Orofacial Function

Orofacial function was evaluated using the Nordic Orofacial Test Screening tool (NOT‐S) [22]. This assessment consists of a structured interview and a clinical examination, each covering six functional domains. The interview evaluates sensory, respiratory, masticatory, and salivary functions, while the clinical examination assesses facial expressions, nasal breathing, and oral motility. Each domain is scored to establish an overall score, which serves to assess the severity of dysfunctions.

### 2.3 Dental Health

All clinical examinations were performed with the aid of dental magnifying loupes (Carl Zeiss, magnification 4.3x) and a frontal light. The presence of dental pathologies was evaluated using the Decayed Missing and Filled Teeth (DMFT) index [23], and oral hygiene using the Plaque Index [24]. The DMFT index was used as a dental health indicator, quantifying the number of decayed, missing, and filled permanent teeth. The Plaque Index was used to assess dental plaque accumulation, with a score ranging from 0 (no plaque) to 3 (severe accumulation), thereby reflecting oral hygiene and the risk of periodontal diseases.

### 2.4 Malocclusions

Finally, the presence and severity of dental malocclusions were assessed through a standard orthodontic evaluation. This included an intermaxillary analysis (measuring overjet, overbite, sagittal molar and canine relationships, the presence or absence of anterior or posterior crossbites, and midline deviation) and an intramaxillary analysis looking at tooth size, arch length discrepancies (noted as an excess or a lack of space), and the depth of the curve of Spee. Overjet was defined as the horizontal distance between the incisal edge of the maxillary central incisors and the labial surface of the mandibular central incisor. Overbite was defined as the vertical overlap between the maxillary and mandibular central incisors. All measurements were millimetric.

### 2.5 Statistical Analysis

All statistical analyses were conducted using SPSS software (version 29, IBM Corp, Armonk, NY, USA). Descriptive statistics were computed for all variables, including means and standard deviations for continuous variables. To evaluate changes occurring during the 12‐month follow‐up period, comparing the two time points (T1 and T2), paired sample *t*‐tests were used for normally distributed continuous variables, including interview scores, examination scores, NOTS scores, Plaque Index, DMFT, overjet, and overbite. Effect sizes were reported using Cohen's d and Hedges’ correction to assess the magnitude of differences.

For categorical variables, such as changes in dental status and oral hygiene habits, chi‐square tests were performed to determine associations between pre‐ and post‐follow‐up evaluations. When expected cell counts were low, appropriate corrections (i.e., Fisher's exact test) were applied. A significance level of *p* < 0.05 was set for all statistical tests.

Comparisons were also made between patients with primarily the bulbar type of ALS and those with the spinal type, to assess whether there was a difference in the evolution of the outcomes between T1 and T2, using independent sample *t*‐tests. Finally, multiple linear regression analyses were carried out to assess whether general disease progression as an independent variable (with other independent variables being ALS type, age, and sex) was a factor associated with the progression of orofacial function, dental health, or malocclusion severity.

## 3 RESULTS

### 3.1 Sample

From the initially included thirteen patients with ALS (10 with primarily the spinal type and 3 with the bulbar type), three passed away (two with the spinal and 1 with the bulbar type) and were thus lost to follow‐up. Ten patients were therefore seen at the two‐time intervals (baseline and 12 months afterwards).

### 3.2 General Disease Course

**FIGURE 1:**
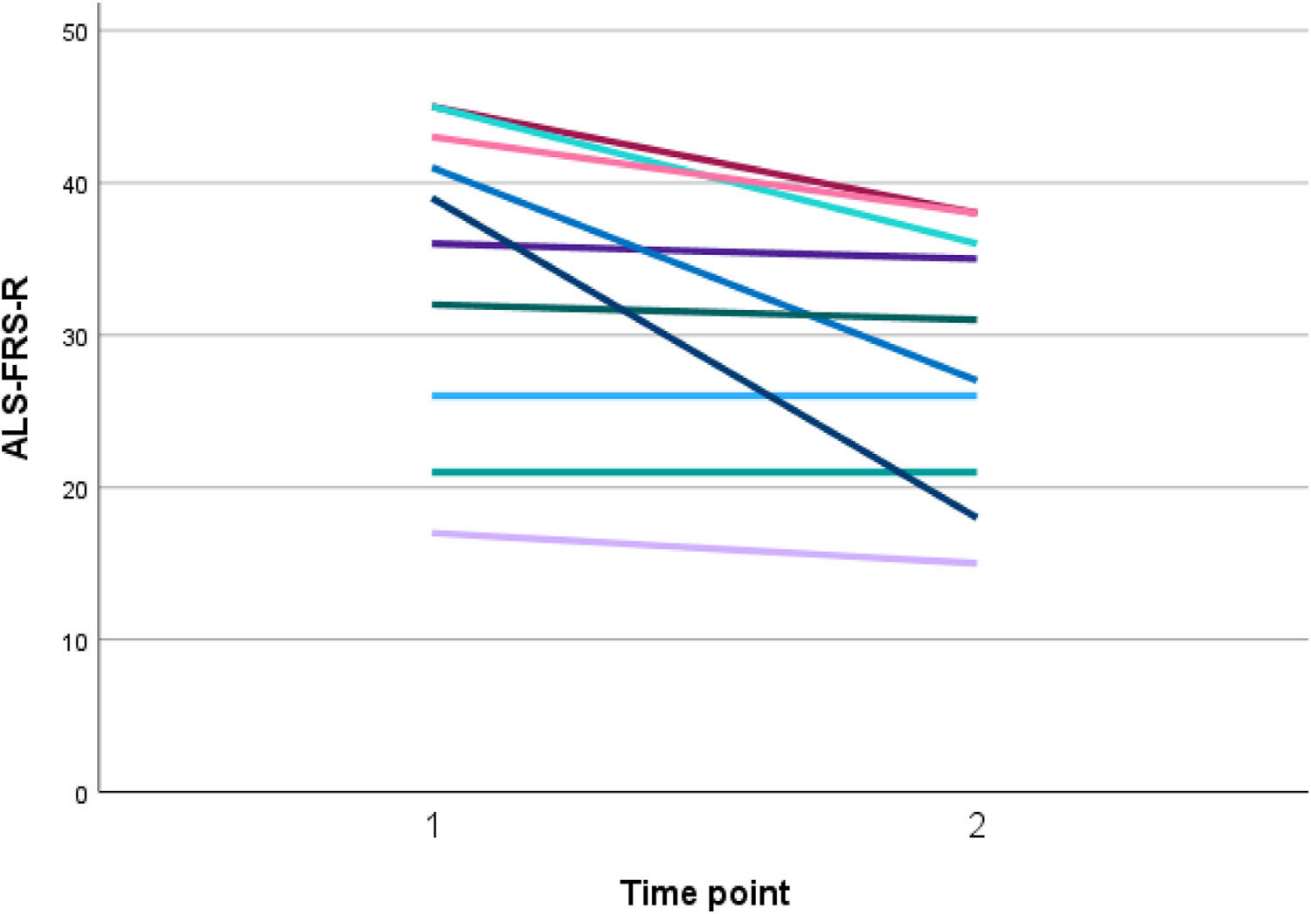
General disease course over the 12‐month period, from T1 (baseline) to T2 (one year later), showing the decline in ALS Functional Rating Scale Revised (ALS‐FRS‐R) score for all of the 10 longitudinally‐followed patients.

A significant deterioration was observed concerning the general disease course (Table 1). The mean ALS‐FRS‐R scores worsened significantly from T1 = 34.50 ± 10.14 to T2 = 28.50 ± 8.45 (Figure 1) with a mean difference of 6 ± 6.98 (95% CI = 1.01, 10.99; *p* = 0.024).

**TABLE 1:** Evolution of general disease course (using the ALS Functional Rating Scale Revised score—AS‐FRS‐R), orofacial function (using the Nordic Orofacial Test Screening tool—NOTS), and dental health (using the Decayed, Missing, and Filled Teeth index—DMFT; and the Plaque Index) over the 12‐month experimental period from T1 (baseline) to T2 (one year later). Means and standard deviations are shown, and p‐values refer to differences between T1 and T2 scores.

	ALS‐FRS‐R score	NOTS total score	NOTS interview score	NOTS examination score	DMFT score	Plaque Index
T1 (Mean ± SD)	34.5 ± 10.1	3.9 ± 2.3	1.9 ± 1.3	2.0 ± 1.5	19.0 ± 7.9	1.6 ± 0.7
T2 (Mean ± SD)	28.5 ± 8.4	6.2 ± 2.9	2.9 ± 1.8	3.3 ± 1.6	20.8 ± 8.5	2.0 ± 0.7
*p*‐value	0.024	0.001	0.0023	0.004	0.014	0.004

### 3.3 Orofacial Function

**FIGURE 2:**
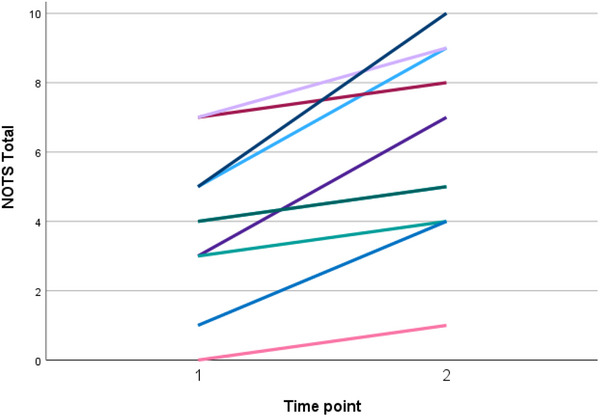
Orofacial function over the 12‐month period, from T1 (baseline) to T2 (one year later), showing the increase in the Nordic Orofacial Test Screening tool (NOTS) total score (indicating a decline in orofacial function) for all of the 10 longitudinally‐followed patients.

A significant deterioration was observed in orofacial function (Table 1), as assessed by the NOTS Total score, which increased by an average of 2.3 points (*p* = 0.001), indicating a worsening of neuromuscular and orofacial dysfunctions over time (Figure 2). Similarly, both interview and examination scores worsened significantly, with mean increases of 1.0 point (*p* = 0.023) and 1.3 points (*p* = 0.004), respectively, from T1 to T2, suggesting a progressive decline in clinical assessment outcomes.

### 3.4 Dental Health

**FIGURE 3:**
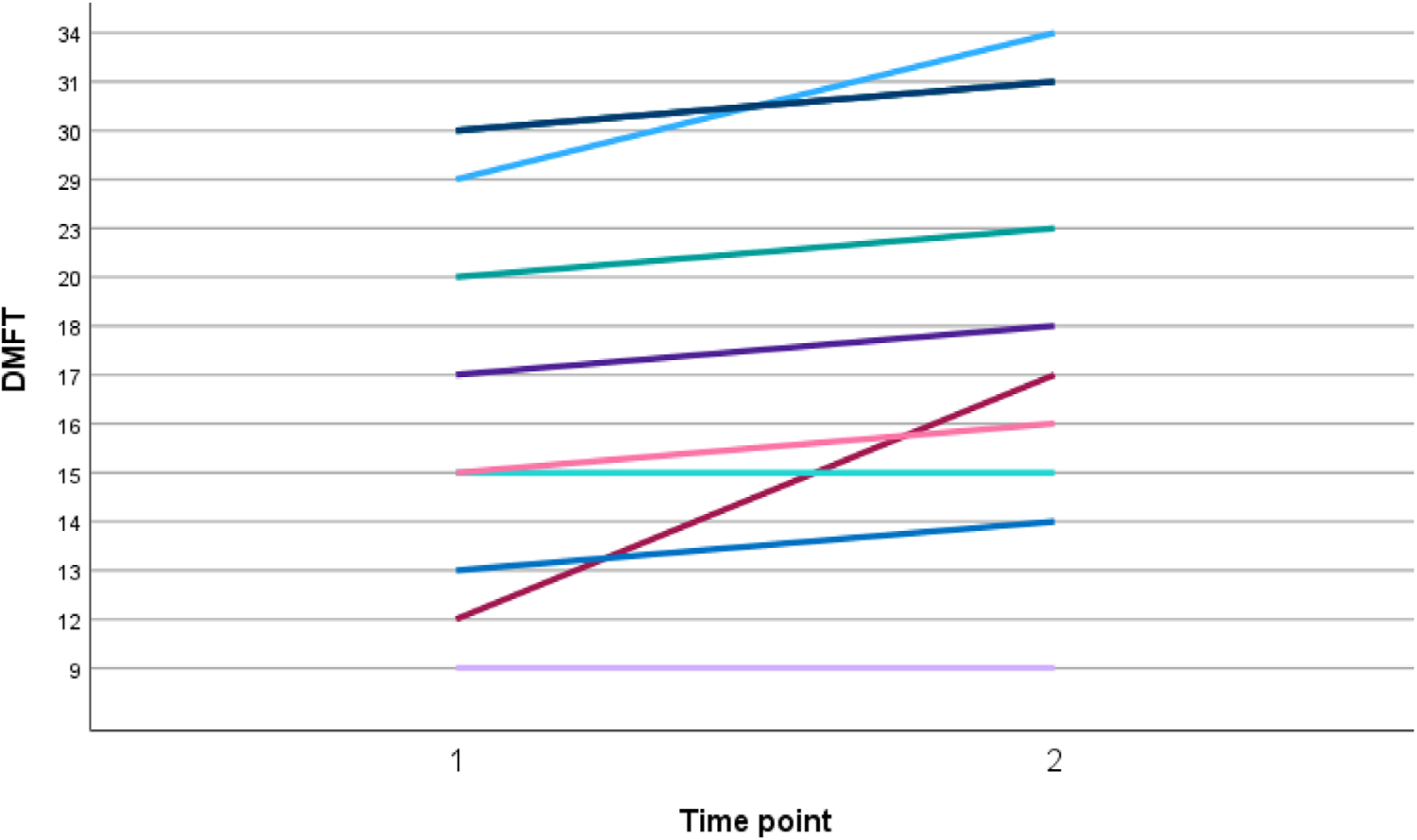
Dental health over the 12‐month period, from T1 (baseline) to T2 (one year later), showing the increase in the Decayed Missing and Filled Teeth (DMFT) score (indicating an increase in the number of affected teeth) for all of the 10 longitudinally‐followed patients.

General oral health also showed a significant deterioration from T1 to T2 (Table 1). The DMFT score increased on average by 1.8 additional teeth (*p* = 0.014), reflecting an increase in caries, missing, or restored teeth over time (Figure 3). Additionally, the Plaque Index rose by 0.4 points (*p* = 0.004), indicating a worsening of oral hygiene and plaque accumulation.

Chi‐square tests conducted on categorical variables further supported these findings. Although changes in dental status showed a trend towards a decline (*p* = 0.065), these changes did not reach statistical significance. Similarly, shifts in oral hygiene habits followed the same pattern, with a slight but non‐statistically significant change (*p* = 0.068), indicating that while some participants exhibited worsening hygiene behaviors, the overall trend remained inconclusive.

### 3.5 Malocclusions

Dental occlusion parameters remained stable throughout the study period. No significant changes were observed in overjet (*p* = 0.860) or overbite (*p* = 0.347), suggesting that despite the deterioration in oral health, occlusal relationships were not notably affected within the time frame of the analysis.

### 3.6 Associations

ALS type only seemed to be associated with the progression of the NOTS total score from T1 to T2, with those having the spinal type showing worse progression than those with the bulbar type (2.63‐point increase versus 1‐point increase, respectively; *p* = 0.024).

When assessing associations between general disease progression (along with ALS type, sex, and age as independent variables) with the progression of orofacial function, dental health, or malocclusion severity, the only significant regression model was for the changes in the NOTS examination score from T1 to T2 (*R* ^2^ = 0.885; *p* = 0.014). Within this model, general disease progression was positively associated with worsening in the NOTS examination score (p = 0.005), and the bulbar type of ALS was also associated with worsening in the NOTS examination score (*p* = 0.020).

## 4 DISCUSSION

The results of the present study highlight a progressive deterioration of orofacial function in patients with ALS, characterized by a significant increase in orofacial dysfunction (as assessed by the NOTS) and worsening dental health, as evidenced by elevated DMFT scores and Plaque Index, but without any remarkable changes in the stability of occlusal parameters. The bulbar type of ALS seems to show worse deterioration of orofacial function over time, and individuals with more significant general disease progression also show worse orofacial functional decline. This strengthens the hypothesis that functional deterioration in ALS follows a neuromuscular pattern that precedes structural occlusal changes.

This progression is consistent with previous studies documenting similar functional deterioration. For instance, Riera‐Punet et al. (2018) [9] reported a reduction in masticatory function and deterioration of oral health status in patients with ALS, primarily due to oral muscle weakness and difficulties in maintaining adequate oral hygiene. Similarly, Nakayama et al. (2018) [8] emphasized the impact of dysphagia on dental health, explaining that the inability to chew properly and maintain sufficient salivation promotes the development of caries and periodontal disease. These findings are further supported by the work of Bergendal and McAllister (2017) [10], who stressed the importance of tailored dental care throughout disease progression, given the increasing difficulties patients face in maintaining oral hygiene due to loss of autonomy.

However, contrary to expectations based on other degenerative neuromuscular diseases, such as Duchenne muscular dystrophy, where early occlusal alterations are frequently observed, this study shows that the occlusal parameters of patients with ALS remain relatively stable. This suggests that occlusal changes in ALS are more limited and occur later, likely due to the adult‐onset nature of the disease and progressive tooth loss associated with feeding difficulties and reduced mobility. These conclusions align with previous observations [13], which showed that malocclusions in ALS are less pronounced due to the progressive deterioration of the dentition and the minimal impact of the disease on the growth and development of orofacial structures in adults.

Nevertheless, the increase in DMFT scores and Plaque Index, combined with the stability of occlusal parameters, underscores the importance of regular dental follow‐up and preventive management to limit oral health complications and improve the quality of life of patients with ALS. As the loss of autonomy progresses, an interdisciplinary approach involving neurologists, dentists, and other healthcare professionals becomes essential to ensure optimal oral health management in this progressive disease.

This study presents several strengths, including its prospective longitudinal design, the use of validated clinical tools (ALS‐FRS‐R, NOTS, DMFT), and the repeated assessment of the same individuals over 12 months. These methodological elements reinforce the internal consistency of the findings despite the challenges inherent to working with a fragile population.

However, important limitations must be acknowledged. The small sample size and the use of a convenience sampling approach, with the sample drawn from all eligible patients with ALS able to participate during the recruitment period, reduce statistical power, increase the likelihood of selection bias, and restrict the generalizability of the findings. The monocentric nature of the study further limits the generalizability of the findings to the broader ALS population, particularly given the heterogeneity of the disease. Moreover, the limited follow‐up period may have reduced the ability to detect long‐term changes, particularly in occlusal development, although a longer follow‐up period may have led to the unfortunate loss of more patients.

The absence of a control group also limits comparison with unaffected individuals. Selection bias may have occurred, as participants were recruited from a single center and likely had better baseline autonomy. Furthermore, potential confounders such as medication use, diet, and caregiver support were not controlled for and may have influenced oral health outcomes. Lastly, clinical assessments were performed in various settings (clinic, hospital, or at home), which could have introduced measurement variability despite standardized protocols. The recording of the occlusion on dental casts or dental scans could possibly offer a more precise evaluation of subclinical changes that may have occurred. Nevertheless, the clinical situation of the patients did not allow us, from an ethical perspective, to expose them to the extra inconvenience of dental impression taking.

## 5 Conclusions

ALS seems to have a progressive and significant impact on orofacial function and oral health, as evidenced by the worsening of NOTS scores, DMFT scores, and plaque accumulation over time. The progressive loss of autonomy associated with the disease likely contributes to this deterioration, making oral self‐care increasingly challenging for affected individuals.

The absence of significant changes in dental occlusion parameters suggests that structural relationships remain stable, but the decline in oral hygiene and increased disease burden highlight the urgent need for proactive and lifelong oral health management in patients with ALS. Given the severe systemic impact of ALS, preserving oral health is crucial to maintaining quality of life and minimizing additional complications in an already vulnerable population.

Following on from these findings, several priorities for improving patient care may be suggested:

- Implementing early, regular, and preventive dental follow‐up to compensate for reduced self‐care capacity;
- Supporting caregivers through clear oral‐hygiene protocols adapted to functional decline;
- Anticipating common complications, such as sialorrhea, xerostomia, reduced mouth opening, and aspiration risks;
- Encouraging measures that help maintain residual orofacial function, including exercises targeting tongue mobility, lip seal, or masticatory capacity when appropriate;
- Focusing on maintaining comfort, nutrition, and oral cleanliness, recognizing their direct impact on general health and quality of life.

Overall, the present findings may help clinicians become aware of the importance of structured, anticipatory, and coordinated oral‐health management in ALS, aiming to preserve oral function, prevent complications, and support the well‐being of patients facing progressive neuromuscular decline.

## Author Contributions

**Vincent Vaudroz**: Investigation, data curation, formal analysis, writing – original draft. **Stavros Kiliaridis**: Conceptualization, methodology, writing – review & editing. **Annemarie Hübers**: Methodology, writing – review and editing. **Gregory S. Antonarakis**: Conceptualization, methodology, writing – review and editing, supervision.

## Funding

The authors received no funding for the present study.

## Conflicts of Interest

The authors declare no conflicts of interest.
